# Initial Clinical Trial of Robot of Endovascular Treatment with Force Feedback and Cooperating of Catheter and Guidewire

**DOI:** 10.1155/2018/9735979

**Published:** 2018-04-16

**Authors:** Yuhua Jiang, Keyun Liu, Youxiang Li

**Affiliations:** Department of Interventional Neuroradiology, Beijing Neurosurgical Institute and Beijing Tiantan Hospital, Capital Medical University, Beijing 100050, China

## Abstract

To evaluate the feasibility and safety of the robot of endovascular treatment (RobEnt) in clinical practice, we carried out a cerebral angiography using this robot system. We evaluated the performance of application of the robot system to clinical practice through using this robotic system to perform the digital subtraction angiography for a patient who was suspected of suffering intracranial aneurysm. At the same time, through comparing the postoperative head nuclear magnetic and blood routine with the preoperative examination, we evaluated the safety of application of the robot system to clinical practice. We performed the robot system to complete the bilateral carotid artery and bilateral vertebral arteriography. The results indicate that there was no obvious abnormality in the patient's cerebral artery. No obvious abnormality was observed in the examination of patients' check-up, head nuclear magnetism, and blood routine after the digital subtraction angiography. From this clinical trial, it can be observed that the robot system can perform the operation of cerebral angiography. The robot system can basically complete the related observation indexes, and its accuracy, effectiveness, stability, and safety basically meet the requirements of clinical application in neurointerventional surgery.

## 1. Introduction

With lifestyle changing, the population of “hypertension, hyperlipidemia, and hyperglycemia” is increasing year by year. The incidence of cardiovascular and cerebrovascular disease is increasing. At present, the morbidity, recurrence rate, and mortality of cardiovascular and cerebrovascular disease are very high [1, 2]. Endovascular treatment is a safe, effective, minimally invasive new method to be promoted in clinical treatment. It plays an increasingly important role in the treatment of vascular disease and gradually becomes the best treatment, while there are still some bottlenecks restricting the development of the endovascular treatment. For example, high demand of operation technique, stability and accuracy, radiation exposure, and long-term radiation exposure of vascular interventional surgeon may lead to leukocyte reduction and cell carcinogenesis, and so on. At the same time, there is a limit to the stability and precision of human hand operation.

Despite technology is progressing in vasointerventional materials and vascular intervention techniques, many of these procedures continue to be several hours in duration and require the use of significant amounts of fluoroscopy resulting in considerable radiation exposure to the vascular interventional surgeons [3, 4]. Although protective garments can protect parts of the vascular interventional surgeon's body from radiation scatter, such protective garments are heavy and uncomfortable resulting in increased surgeon fatigue during long procedures as well as physical stresses that will result in orthopedic injuries [5, 6]. And fatigue can reduce the stability and accuracy of surgical operations.

But the vascular intervention robot conforms to the clinical requirements. Robots have the following advantages: high accuracy, stability, and safety; the interventional surgeon can operate the robot outside the operating room, and the radiation damage is small. Other systems for vascular interventional surgery have been developed to the degree that allows the vascular interventional surgeon to control and direct catheter movement at a distance from the patient [7–13]. These systems allow the vascular interventional surgeon to be removed from radiation exposure without the use of cumbersome protective garments. However, these systems are expensive and complex, and the product positioning is relatively unitary, it can only be used for the single type of vascular disease, but not various types of vascular disease.

In view of the above deficiencies, cooperating with the Beijing Institute of Technology, we have developed a master-slave cardiovascular and cerebrovascular interventional surgical robotic system—the robot of endovascular treatment (RobEnt). The robot system increases the accuracy of force feedback measurement, improves the accuracy of catheter push and rotation, reduces the damage to the surface of catheter and guidewire, and increases the true sense of operation at the same time. And the complex operation can be accomplished by the cooperative operation of the catheter and guidewire. Meanwhile, the system adopts master-slave operation mode, the master controller is located outside the operating room, and the slave manipulator is set in the operation room. Through the doctor operates the handle of the master controller, the control signal is transmitted to the slave, using the slave manipulator to complete the operation. By using master-slave operation, the doctor can avoid the operation directly under the radiation line and solve a series of problems caused by the long-term acceptance of radiation.

The robot system is equipped with a catheter controller and a guidewire controller on the slave end device, so that the catheter and the guidewire can be operated simultaneously, and it has tactile feedback system. The surgeon can feel the resistance of the catheter and the guidewire on the operating handle. It solves the problem that the existing robot is difficult to complete the cooperative operation of the catheter and guidewire and cannot intuitively feel the operative procedure. The slave manipulator takes the form of multiple platform-connecting blocks on one track; the platform-connecting blocks are used for fixing the catheter controller or guidewire controller and control the axial movement of the catheter and guidewire on a single track. Due to the use of a plurality of blocks running on the same track, running distance of each slider coincides. It can improve the safety and operability of interventional operation or angiography, and the structure is simple and easy to implement. The device can also be used for surgical simulation and training.

The robot system can perform multiple vascular interventional operations including cerebral arteriography, angiography of the heart, renal arteriography, and so on. At present, the robot system has completed the performance self-test and 10 animal experiments. The analysis of the experimental process and results indicated that the robot achieved the same operation quality as manual operation. Furthermore, we conducted a clinical trial using the robot system to verify its feasibility and safety applied to clinical practice. This report describes the vascular intervention robotic system's performance and safety in this clinical study.

## 2. Materials and Methods

### 2.1. Patient

Five inpatients aged from 20 to 60 years old were chosen in our hospital. All the patients were suspected of having intracranial aneurysms and needed to be determined by cerebral angiography. The exclusion criteria included the patients who were long time in bed, could not take care of oneself, could not cooperate with inspection, used antibiotics in two weeks, suffered from serious internal medicine diseases, chronic inflammatory diseases, acute cerebrovascular events (included ischemic stroke, cerebral hemorrhage, and subarachnoid hemorrhage) in six months, other cerebrovascular diseases, brain tumor, and other known neurological diseases, and could not sign informed consents.

In the end, we screened out the most appropriate patient according to exclusion criteria and after the contrast analysis. The participating patient gave informed consent for study participation. The study protocol was approved by the local ethics committee.

According to the requirements of preoperative examination of cerebral angiography in our hospital, we had carried out the related examinations, including chest X-ray, electrocardiogram, blood routine, various biochemical tests, and so on. Inspection results were diagnosed by two specialist neurointerventional surgeons under the double blind. They agreed that the patient had no contraindications for digital subtraction angiography (DSA), and the patient should have a DSA to make a clear diagnosis.

### 2.2. Experimental Instrument

1. The robot of endovascular treatment mainly consists of two parts: the master controller console and the slave manipulator.
2. Conventional surgical equipment and vascular intervention equipment and consumables are presented. The main consumables are as follows:
  - Femoral artery sheath module, 5F, DQ05113818, Y connector assembly, 5F, DMK-Y (Beijing Demax Medical Technology Co. Ltd.)
  - Angiographic catheter, 5F, head angle 135°, 451-514HO (Cordis Corporation)
  - 0.035-inch hydrophilic membrane guidewire, 150 cm, RF^∗^GA35153M (Terumo Corporation)
  - Medical angiography X-ray machine, Artis zee III floor (Siemens)
3. Drugs and reagents are the following:
  - 2% lidocaine hydrochloride injection (Beijing Yimin Pharmaceutical Co. Ltd.)
  - 5% glucose injection (Shanghai Baxter Healthcare Ltd.)
  - 0.9% saline injection (Shanghai Baxter Healthcare Ltd.)
  - Iohexol injection (Shanghai AXA Pharmaceutical Co. Ltd.)
  - Low-molecular-weight heparin calcium injection (GlaxoSmithKline (Tianjin) Co. Ltd.)

### 2.3. Experimental Procedure

**Figure 1:**
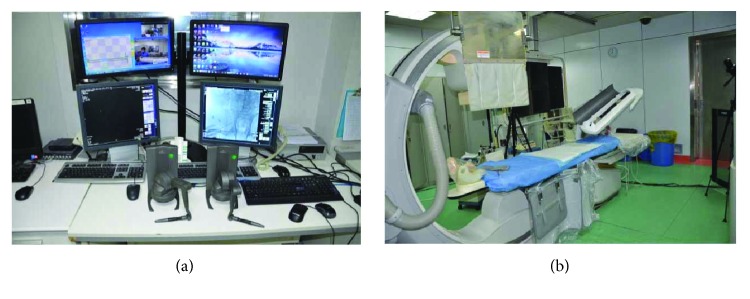


**Figure 2:**
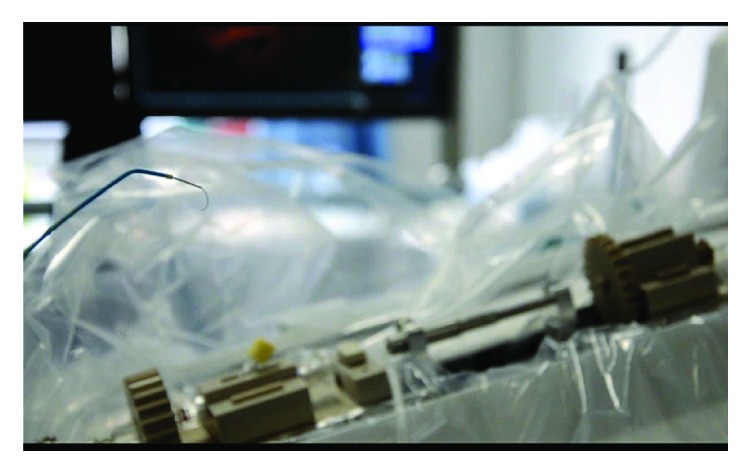


**Figure 3:**
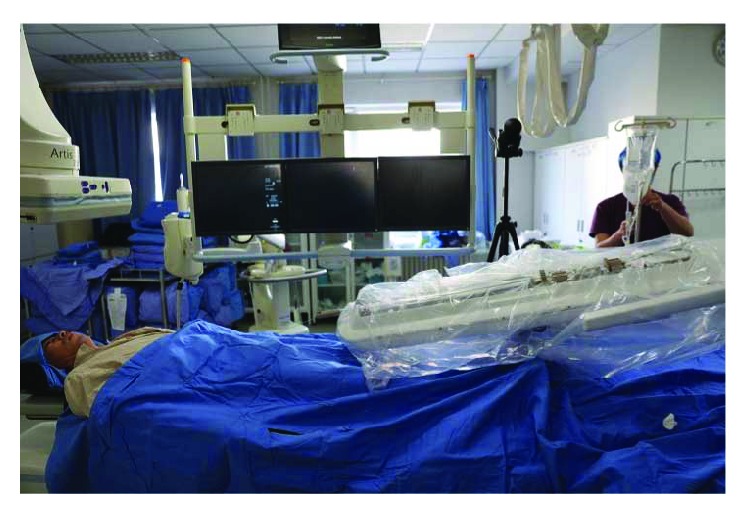


**Figure 4:**
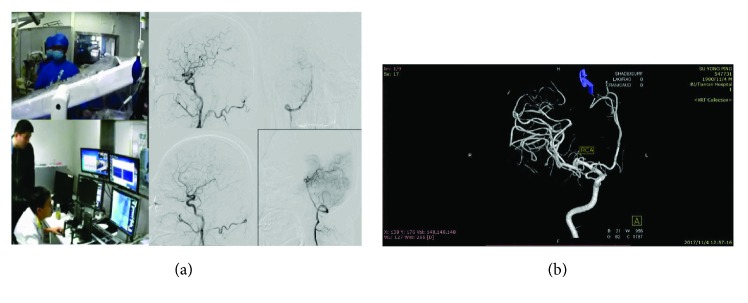


**Figure 5:**
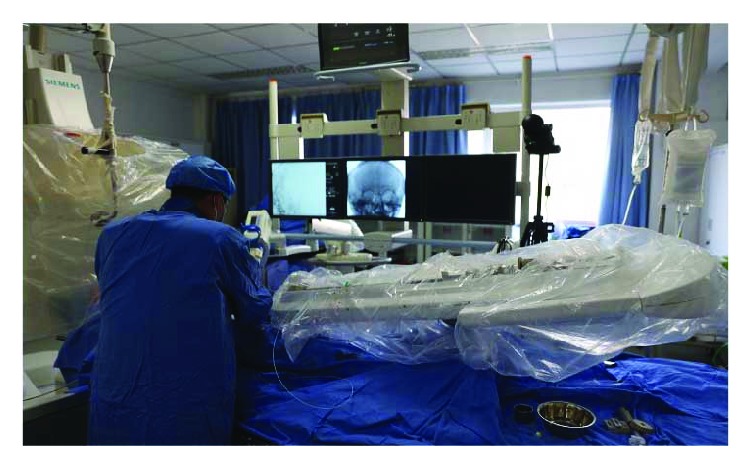


1. After disinfection and sterilization, the robot system was installed in the operating room. The master controller console and the slave manipulator are fixed, respectively. We adjusted the angle of the slave manipulator and operated the handle of the master controller console. The performance of RobEnt was satisfactory (Figure 1).
2. We connected the Y type valve assembly, 5F single bend angiographic catheter, and 150 cm hydrophilic membrane guidewire, respectively, to the system and operated the control platform handle to control the guidewire and catheter forward, backward, and rotation. The guidewire and catheter movement performance was good (Figure 2).
3. The bilateral inguinal surgery area was disinfected with 0.5% iodophor for two times, and the routine operation sheet was given. The 5F femoral artery sheath was inserted into the femoral artery at the left side of the patient, and the Seldinger puncture was performed. 5F single bend angiographic catheter was injected with high-pressure heparin saline continuously, and the catheter was inserted into the artery sheath about 5 cm (Figure 3).
4. Adjusting the operating bed and making the guidewire and catheter exposed to the X-ray, the surgeon controlled the operation handle at the operating table, keeping the catheter position unchanged, advanced guidewire about 10 cm. X-rays showed that the guidewire had entered the left iliac artery; we rotated the guidewire tip pointing to the left, keeping the relative position between the guidewire and catheter unchanged, meanwhile advanced the guidewire and catheter. The guidewire and catheter were sent into the aortic arch along the abdominal aorta. Keeping the catheter position unchanged, the surgeon controlled the guidewire into the right carotid artery and pushed the catheter along the guidewire, then selected the appropriate position, withdrew the guidewire, and performed the right carotid artery angiography. The right vertebral artery, the left common carotid artery, and the left vertebral artery angiography were performed, respectively, and the three-dimensional angiography was performed on the right common carotid artery. The cerebral angiography showed good, and there was no obvious abnormality (Figure 4).
5. After the angiography, we pulled out the catheter and femoral artery sheath. The puncture site was compressed for 15 minutes, pressure dressing on the puncture point (Figure 5).
6. After withdrawing the robot system from the operating room, the catheter room was cleaned, and related disinfection was carried out.
7. After surgery, the changes of the patient's condition were closely monitored, and the blood routine and the head nuclear magnetism were reviewed.

## 3. Results

**Figure 6:**
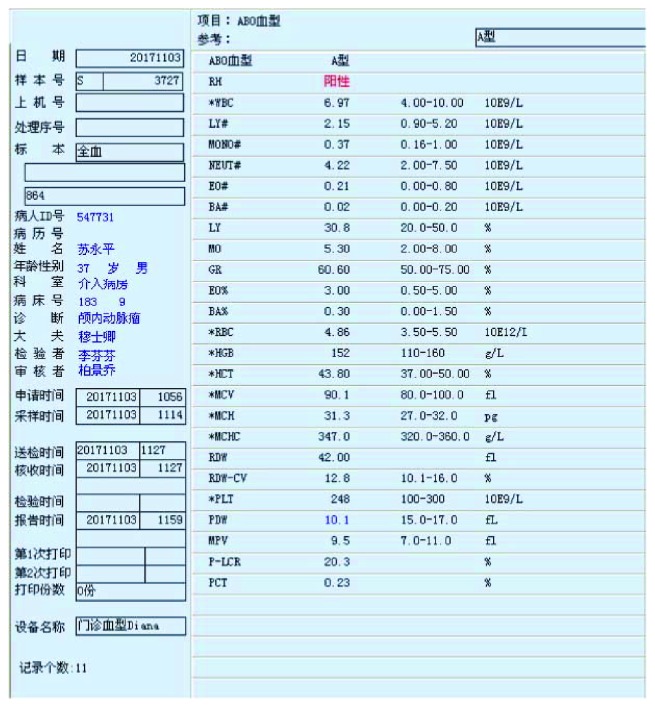
Preoperative blood routine examination.

**Figure 7:**
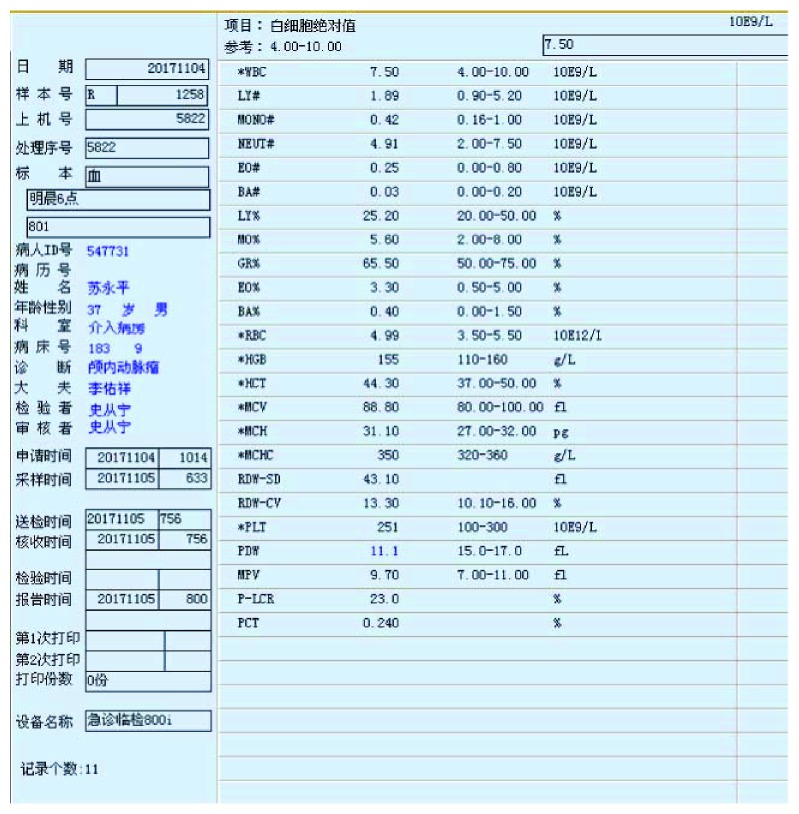
Postoperative blood routine examination.

**Figure 8:**
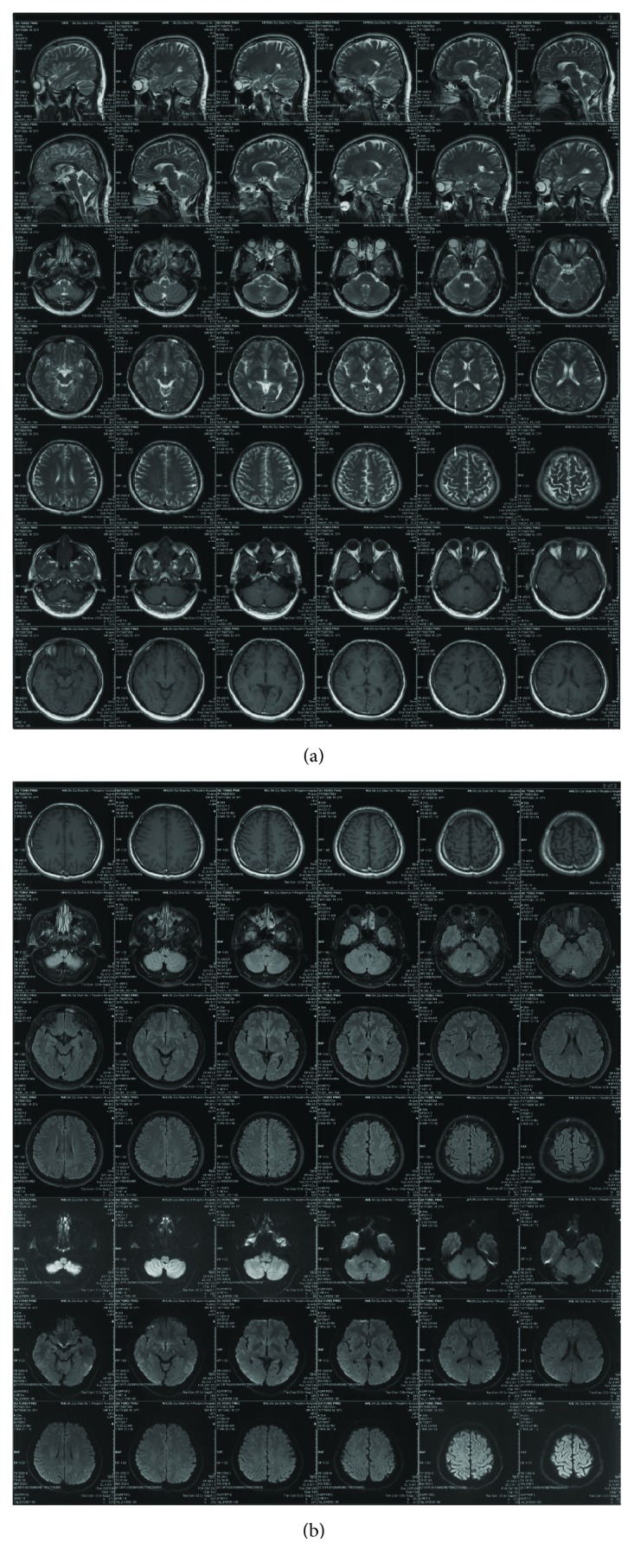
Preoperative head nuclear magnetic resonance examination.

**Figure 9:**
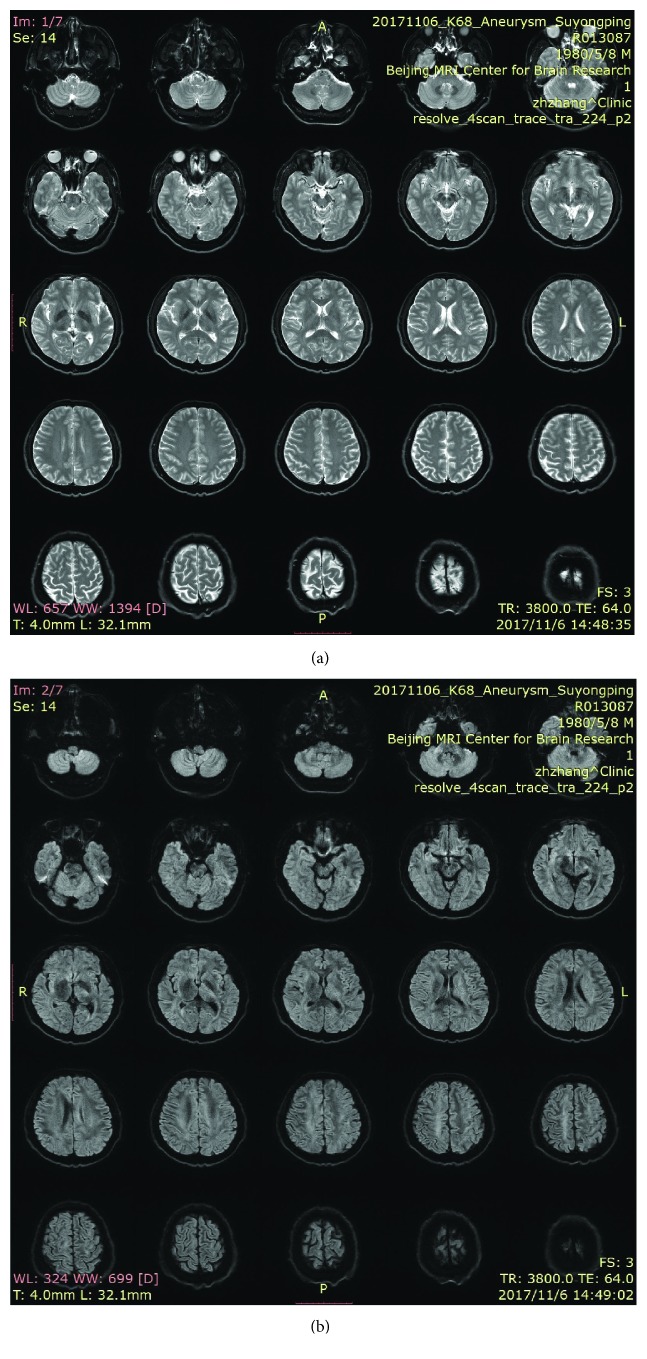
Postoperative head nuclear magnetic resonance imaging.

1. Accuracy, effectiveness, and stability index are the following:
  - The surgeon's console basically meet ergonomic and can provide the operating platform in line with the surgeon's operating habits.
  - The master controller console can monitor the operation information of the catheter and guidewire (including microwire and microcatheter), the resistance condition of the catheter and guidewire, the position of the catheter and guidewire, and the contact of the catheter and guidewire with the blood vessel.
  - The handle of the console is flexible and reliable, which can provide the force feedback for the operator. The doctor can feel the catheter state during the operation and carry out the operation according to the force information of the catheter.
  - When the gear held the guidewire and the catheter, it had a good fit and will not damage the surface coating of the catheter and guidewire.
  - The robot system can maintain the synchronization action with the doctor-operating lever.
  - The robot system does not affect the operation of the equipment around the operating bed after installation and has good operability.
  - Under the X-ray operation, the system can accurately and timely realize the movement of the single curved angiographic catheter and 150 cm super slide loach guidewire in the blood vessel forward, backward, and rotation, consistent with the master and the slave, and can accurately and timely reflect the operation of the master and the slave, and had achieved the following indicators:
    - The maximum movement distance of the catheter and the guidewire is 0.9 m, the positioning accuracy of the axial position is not less than 0.2 mm, and the rotation accuracy is not less than 2 degrees. The maximum speed of linear motion can reach 90 cm/s, and the maximum rotation speed can reach 420°/s.
    - The sterilization time of robot system is less than 1 h.
    - The time of changing the catheter and the guidewire is less than 3 min.
    - The DSA device will not affect the stability of the robot system.
    - The robot system can control the catheter and guidewire into any branch vessel with an inner diameter greater than 3 mm and an angle less than 90 degrees from the main vessel.
  - From the slave manipulator, the catheter can be placed into the patient's blood vessel accurately and timely according to the doctor's instructions.
2. Security index is as follows:
  - After the whole brain angiography was carried out with the robot system, the elastic bandage of the puncture site of the patient was bandaged for 24 hours. The puncture site had no oozing of blood and exudate, and there was no swelling, heat pain, and other infection signs. After 24 hours, the patient can walk out of bed without discomfort symptoms.
  - The time of complete cerebral angiography using the robotic system was about 40 minutes, which was almost the same as that of the interventional surgeons in our hospital.
  - Routine blood test is as follows:
  - The patient underwent routine preoperative examination after admission. There was no obvious abnormality in blood routine. Blood routine examination was repeated 24 hours after operation, and no obvious abnormality was found. Moreover, the patient is generally in good condition, no fever, and other signs of discomfort (Figures 6 and 7).
  - Head nuclear magnetic examination is as follows:
  - Intracranial aneurysm has been suspected after head MRI in the local hospital of the patient, and then the suspected has been overturned in our hospital after performing cerebral angiography. 48 hours after the operation, we performed 7.0 T nuclear magnetic resonance examination on the head of the patient in the Biophysics Institute of Chinese Academy of Sciences. The results showed that there were no new ischemic or hemorrhagic foci after the diagnosis of two interventional surgeons, and there was no obvious abnormality in the cerebral vessels (Figures 8 and 9).

## 4. Discussions

This clinical trial demonstrated that the robot system has the ability to complete cerebral angiography. The important precondition for complete cerebral angiography is to super select the guidewire and catheter to enter each branch of cerebral artery. In this clinical trial, we successfully operated the robot to super select the catheter and guidewire into the main cerebral arteries. The operating time of the robot system was not significantly different from that of the interventional surgeons in our hospital. The speed and accuracy of the advancing, retreating, and rotating of the guidewire and the catheter can be continuously adjusted by the slave manipulator, and the catheter and the guidewire can cooperate with each other and deliver simultaneously. The feasibility, accuracy, and stability of the robot system are up to standard. Compared with hand operation, the robot system has better accuracy and stability and meets the requirements of clinical vascular interventional surgery.

Three aspect inspections have been used to estimate this system's security.

First, the femoral artery puncture site may produce additional damage by the process that the slave manipulator holds the guidewire and the catheter into the femoral artery through the femoral artery sheath, and the damage is mainly estimated by physical examination and the symptoms of the patient. After the operation, the puncture site has no errhysis, no seepage, no inflamed hot pain, and so on, and no masses have been found by palpation and no vascular murmur by auscultation. The patient can get off of the bed freely by himself 24 hours later after the operation, with no activities and sensory disturbance. Therefore, in this cerebral angiography, the robotic system did not cause additional damage to the puncture artery.

Secondly, another indicator to evaluate safety of the system is the effect of aseptic operation, mainly observing whether or not the patient has an infection after surgery. The robot system was installed in the surgical bed in a sterile environment after being strictly disinfected, and the sterile membrane had been used to cover the machine before operation. The contact area of the slave manipulator between the guidewire, catheter, and the femoral artery sheath was strictly disinfected again. Before operation, the patient had no fever, no abnormal blood routine, and no related infections. 24 hours later after the operation, there was no abnormality in the blood test. Vital signs had been checked every 12 hours after operation, and no fever or other discomforts occurred when the patient was discharged on the third day. It is indicated that this robot system is qualified for the aseptic operation of total cerebral angiography.

Finally, the most serious complication of cerebrovascular angiography is cerebrovascular events, and the occurrence of cerebrovascular events is an important index to evaluate the operation safety of the robot. Therefore, we have been completed the examination of head 7.0 T MRI for the patient 48 hours later after operation. The results of the cranial MRI were viewed by two professional neurointerventional surgeons; combined with the head MRI before operation, new focal ischemia or hemorrhage of intracranial had not been found. At the same time, 24 hours and 48 hours later after operation, the positive signs associated with cerebral vascular events of the patient had not been found, such as dizziness, headache, physical activity disorder, and sensory dysfunction. On the seventh day after the operation, we conducted a telephone follow-up to the patient, and the patient had no positive signs of the nervous system. This indicates that there are no related cerebral vascular complications in this operation of the robotic system for cerebral angiography. And this robot system is safe to be used in clinic.

In recent years, vascular interventional surgery has developed rapidly, and the progress of related technologies and the upgrading of supporting equipment have enabled interventional surgeons to perform more complicated vascular interventional procedures. However, because the accuracy and stability of hand operation are not enough, there is still a high risk of complex surgery. At the same time, the number of vascular interventional procedures is increasing, and the total operation time is also increasing. The amount of X-ray radiation and the strain caused by wearing protective equipment to the body also increased [3–6]. This mode of operation that the main end system outside the operating room manipulates the slave end system in the operating room to perform vascular interventional operation had successfully solved these problems. It allows the operator to perform vascular interventional procedures without radiation protection on the outside of the radiation field and has higher accuracy and stability.

At the same time, the robot system is equipped with a catheter controller and a guidewire controller on the slave end device, which can operate the catheter and guidewire simultaneously, and it has the tactile feedback system, which solves the problem that the existing technology is difficult to meet the requirements of the cooperative operation of the catheter and the guidewire and cannot directly feel the tactile information as the traditional surgical operation. It improves the safety and operability of vascular interventional operation, and the doctor can feel the power and achieve more precise operation. The device can also be used for the training of vascular interventional operation. There are other commercial vascular interventional catheter manipulation systems, but they are very different in terms of concept and design [14–17].

There are several deficiencies in this experiment and the robot system. We have only one clinical trial, which cannot be controlled by grouping, and the evaluation of each index of the robot system needs more clinical trials to verify the analysis. Cerebral angiography was performed only in this clinical trial without angiography of other important vessels and organs. Although cerebral angiography requires higher accuracy, stability, and safety, no angiography of other important vessels in the human body may bias the results. The tilt angle of the slave manipulator needs to be adjusted at the time of installation, and it is difficult to adjust the tilt angle in the process of experiment. Therefore, further optimization of the equipment is needed. Because the robot system is in the experimental research and development stage, there is no connection to the DSA device, and experimenter needs to operate DSA equipment under X-ray in the operating room. When this problem is solved, all operations can be performed outside the operating room.

## 5. Conclusions

The result of this clinical trial shows that the robotic system has completed a cerebral angiography with accuracy, stability, and security. The robot system can realize the coordinated operation of the catheter and the guidewire. Meanwhile, the surgeon can perceive the manipulation power and achieve more accurate effect by the equipped force feedback system. We used the robotic system to operate the catheter and guidewire from the femoral artery to the aortic arch and then entered the major cerebral arteries and completed the cerebral angiography. According to the operation effect, the clinical manifestations and examination result of patient, we conclude that the accuracy, effectiveness, stability, and safety of this robot system reach the standard.
